# Identification of bone morphogenetic protein 4 in the saliva after the placement of fixed orthodontic appliance

**DOI:** 10.1186/s40510-021-00364-6

**Published:** 2021-07-12

**Authors:** Lovorka Grgurevic, Ruder Novak, Grgur Salai, Vladimir Trkulja, Lejla Ferhatovic Hamzic, Vojka Zgombic Popovic, Darko Bozic

**Affiliations:** 1 Center for Translational and Clinical Research, Department of Proteomics, School of Medicine, University of Zagreb, Salata 11 Zagreb, Croatia; 2 Department of Anatomy, “Drago Perovic,” School of Medicine, University of Zagreb, Zagreb, Croatia; 3 Department of Pharmacology, School of Medicine, University of Zagreb, Zagreb, Croatia; 4 Orthonova Dental Polyclinic, Zagreb, Croatia; 5 Department of Periodontology, University of Zagreb, School of Dental Medicine, Zagreb, Croatia

**Keywords:** Saliva, BMP4, BMPER, Proteomics, Orthodontic tooth movement, Bone remodeling

## Abstract

**Background:**

This study was conducted in order to explore the effects of orthodontic tooth movement (OTM) on the changes of salivary proteome. This prospective observational pilot study recruited 12 healthy teenage boys with malocclusion treated with a fixed orthodontic appliance and 6 appropriate control participants. Saliva samples were collected a day before and at 0, 2, 7, and 30 days after initialization of treatment, corresponding to the initial, lag, and post-lag phases of OTM. Pooled samples were analyzed by liquid chromatography-mass spectrometry, ELISA, and Western blotting. To date, there is no published data on the presence of BMP molecules or their antagonists in the saliva or in the gingival cervical fluid related to orthodontic conditions.

**Results:**

A total of 198 identified saliva proteins were classified based on their functional characteristics. Proteins involved in bone remodeling were observed exclusively 30 days post appliance placement, including bone morphogenetic protein 4 (BMP4), a BMP antagonist BMP-binding endothelial regulator, insulin-like growth factor-binding protein 3, cytoskeleton-associated protein 4, and fibroblast growth factor 5. Based on the analysis of protein interactions, BMP4 was found to have a central position in this OTM-related protein network.

**Conclusions:**

The placement of a fixed orthodontic appliance induced occurrence of proteins involved in bone remodeling in the saliva at a time corresponding to the post-lag period of OTM. Limitations of this study include a relatively small sample size, limited time of monitoring patients, and the lack of interindividual variability assessment.

**Supplementary Information:**

The online version contains supplementary material available at 10.1186/s40510-021-00364-6.

## Background

Orthodontic tooth movement (OTM) is a mechanically induced process used to treat malocclusion, a misalignment, or incorrect relation between the teeth of the two dental arches. Alveolar bone adapts to the mechanical strains caused by the placement of an orthodontic appliance that is used to correct such malocclusions [1]. In the initial phase, tooth movement depends on the periodontal ligament (PDL), a dense and heterogeneous fibrous connective tissue structure that anchors the teeth to the alveolar bone [2]. It is the most deformable tissue in the periodontium that allows the initial mechanical disruption of the teeth. A lag phase follows when no tooth movement occurs over 4 to 20 days. In this phase, compression of the PDL causes disturbances in its blood flow, leading to cell death and hyalinization [3]. In the post-lag phase, the dead cells are resorbed by resident macrophages and bone remodeling is initiated. Applied mechanical force triggers bone resorption at the pressure side and bone formation at the tension side resulting in tooth movement about 40 days after the initial force application [4]. Numerous inflammatory mediators, cytokines, growth factors, and bone remodeling-related proteins are expressed during OTM; however, the exact mechanism of osteogenesis on the tension side is poorly understood [5].

The salivary proteome has a prominent serum component that is predominantly sourced from the blood vessels of the carotid artery. Most compounds found in saliva are also found in the blood, so changes in the protein composition of the saliva are an indicator of not only local, but also systemic changes that can be associated with certain diseases. One of the major hurdles to the use of the saliva as a diagnostic fluid is its notorious variability in composition: influenced by sex, age, and numerous physiological processes such as pregnancy, exercise and physical activity, cigarette smoking, and stress, as well as inter-individual variation [6, 7]. Salivary proteomics during OTM has been recently reviewed suggesting that several biomarkers could be a useful tool in indicating the effectiveness of orthodontic treatment. Proposed candidates include myeloperoxidase, leptin, and the ratio of nuclear factor kappa B ligand to osteoprotegerin; however, their potential clinical use is overshadowed by inter-individual variation [8]. In terms of novel biomarker discovery by mass-spectrometry-based proteomics, a small number of studies have revealed only a handful of potential markers that could be linked biologically to OTM. Such example is the upregulation of apolipoprotein E, a novel regulator of bone metabolism in mice, induced by orthodontic treatment [9].

Inflammation plays a key role in the remodeling process on the compression side where PDL fibroblasts synthesize proinflammatory cytokines, which stimulate degradation of extracellular matrix and promote cell growth [10]. This aseptic inflammation is essential for highly complex mechanisms of OTM involving transforming growth factor-beta (TGFß) and bone morphogenetic proteins (BMPs) [11]. BMPs belong to the TGFβ superfamily of cytokines with pleiotropic functions in fetal and postnatal development [12]. They were first identified by their ability to induce ectopic bone and cartilage formation, but to date, no BMP molecules have been identified in the saliva. In OTM, it has recently been proposed that the PDL osteocytes, cells responsible for mechanosensing, control alveolar bone remodeling via sclerostin, a potent BMP antagonist [13]. Vascular endothelial cells present in the alveolar bone recruit circulating inflammatory cells to the PDL. This stress-induced inflammatory response is modulated by BMP signaling [14]. Once upregulated, BMP2 and BMP4 induce proinflammatory endothelial phenotype, enhancing leukocyte adhesion to the endothelial surface in vitro [15, 16]. Moreover, mechanical stress induces osteoblast differentiation and an increase in BMP4 gene expression in mouse preosteoblasts and fibroblasts, which suggests that BMP4 could be an important autocrine and paracrine factor in tensile stress-induced osteogenesis [17].

The proteomics-based pilot study was conducted, with intention to screen the potential short-term evaluation (over 30 days) of the human saliva proteome related to the OTM induced by the placement of the orthodontic appliance to treat malocclusion in teenage. Considering their role in the processes encompassing OTM and during tooth root and periodontium formation, it was hypothesized that members of the TGFβ superfamily of cytokines might be detected.

## Materials and methods

### Study participants

**Fig. 1:**
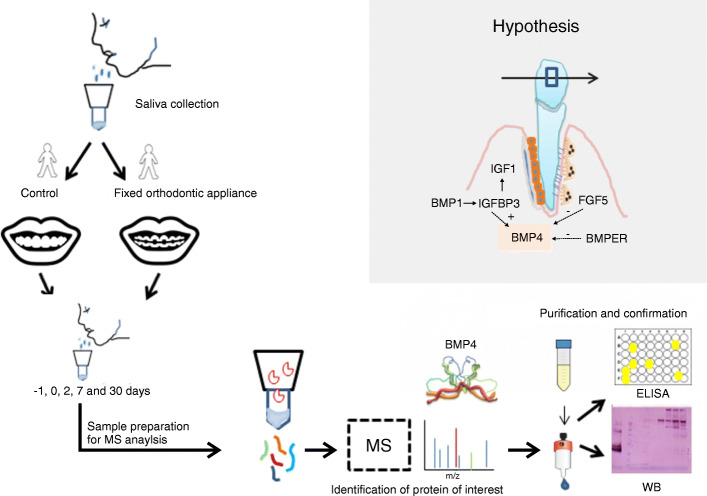
Graphical summary of the conducted pilot study, including the presented hypothesis

This prospective observational pilot study included 18 consecutive patients with malocclusion, admitted at the same orthodontic clinic. The participants met the following criteria: male sex, age of 12 to 14 years, systemically healthy, non-smoker, and indication for MBT orthodontic therapy. Exclusion criteria were local or systemic disease development during the follow-up period, failure to keep follow-up appointments and patients who willingly drop-out from the study; however, no patients were excluded during this pilot study. Male patients with a similar age were chosen in order to minimize the sources of variability and bias of this pilot study. Participants’ individual malocclusion types are presented in the Supplementary table S1. At the beginning of the study, the participants with malocclusions were divided into two groups, 12 cases who underwent orthodontic therapy and 6 controls who did not, for which the Straight wire MBT technique, with a slot 22 and a 0.12 nickel-titanium wire was utilized. For all participants, clinical periodontal parameters were examined by determining the pocket probing depth (PPD), full-mouth plaque score (FMPS), and full-mouth bleeding score (FMPS) [18, 19]. These parameters were measured at six sites per tooth (mesio-buccal, buccal, disto-buccal, mesio-oral, oral, disto-oral aspects) using the UNC 15 mm periodontal probe (Devemed GmbH, Tuttlingen, Germany) and are presented in Table 1. Seven days prior to the commencement of orthodontic therapy, all participants underwent ultrasonic supragingival instrumentation with fine tips only (EMS; Nyon, Switzerland) in order to eliminate gingival inflammation. The study was approved by the Ethics Committee at the School of Dental Medicine (05-PA-15-4/2017). Written informed consent was obtained from all participants and their parents. Study design is graphically depicted in Fig. 1.

**Table 1:** Participants’ clinical periodontal parameters: SD - standard deviation; PPD - pocket probing depth; FMPS - full mouth plaque score, FMBS - full-mouth bleeding score

Variable mean ± SD	Cases	Controls
PPD (mm)	2,30 ± 0.2	2.32 ± 0.3
FMPS (%)	16 ± 3.4	15 ± 3.8
FMBS (%)	14 ± 3.6	13 ± 3.7

### Saliva sampling

Cases’ saliva samples were collected 1 day prior to and then at 0, 2, 7, and 30 days post orthodontic appliance placement corresponding to the initial (0, 2 days), lag (7 days), and post-lag (30 days) phases of OTM. At the same time points, saliva samples were also collected from the 6 controls with malocclusions. Participants refrained from eating and washed their teeth 60 min before saliva sampling. The saliva was expectorated into clean Petri dishes, of which 1 mL was transferred to Eppendorf tubes and immediately frozen at –80°C. For analysis, all individual samples in each group, collected at one time-point were pooled, centrifuged for 10 min at 12 000g at 4°C to remove debris, and proteins were purified by acetone precipitation. Protein concentration was determined using RC DC Protein Assay Kit II (BioRad).

### Mass spectrometry

Pooled protein samples (40 μg) were alkylated (55 mM iodoacetamide in 8 M urea) and digested with 0.8 μg trypsin (Worthington, TPCK treated) in 10-kDa centrifugal filter units. Digested peptides were purified on a pre-column and separated on a C18 nano-column by HPLC (Ultimate 3000, Thermo Fischer Scientific). Mass spectrometry (MS) was performed on an LTQ Orbitrap Discovery instrument (Thermo Fischer Scientific), and raw data was processed using MaxQuant software version 1.5.1.2. (Max Planck Institute of Biochemistry). Proteins were quantified using the intensity-based absolute quantification (iBAQ) algorithm with the false discovery rate at the peptide and protein level set at 1%. Samples were analyzed in technical triplicates, and proteins identified with at least one peptide were considered relevant for analysis [20]. Detected proteins were classified according to their gene ontology, using the European Molecular Biology Laboratory - European Bioinformatics Institute (EMBL-EBI) Quick GO web-based browser [21]. The MS proteomics data were deposited at the ProteomeXchange Consortium via the PRIDE partner repository with the dataset identifier PXD016481. STITCH database version 5.0 (http://stitch.embl.de) was used to analyze interaction networks of proteins functionally classified as bone remodeling proteins (see Supplementary methods 1 for details).

### BMP4 ELISA

Pooled saliva samples obtained 30 days after placement of a fixed orthodontic appliance were applied twice to a heparin Sepharose column (Amersham Pharmacia Biotech). Eluted fractions were precipitated with saturated ammonium sulphate (SAS), and the precipitates were resuspended in phosphate-buffered saline (PBS) [22]. BMP4 protein concentration in wash and eluate solutions was measured in duplicates using BMP4 ELISA DuoSet (#DY314, R&D Systems) according to manufacturers’ instructions (see Supplementary methods 2 for details).

### BMP4 Western blotting

Heparin-enriched proteins from saliva pools were separated by SDS-PAGE and analyzed by Western blotting. rhBMP4 was detected using a mouse monoclonal anti-human BMP4 antibody, available as the capture antibody in the ELISA DuoSet kit (cat. no.#DY314 , R&D Systems) (see Supplementary methods 3 for details).

## Results

**Fig. 2:**
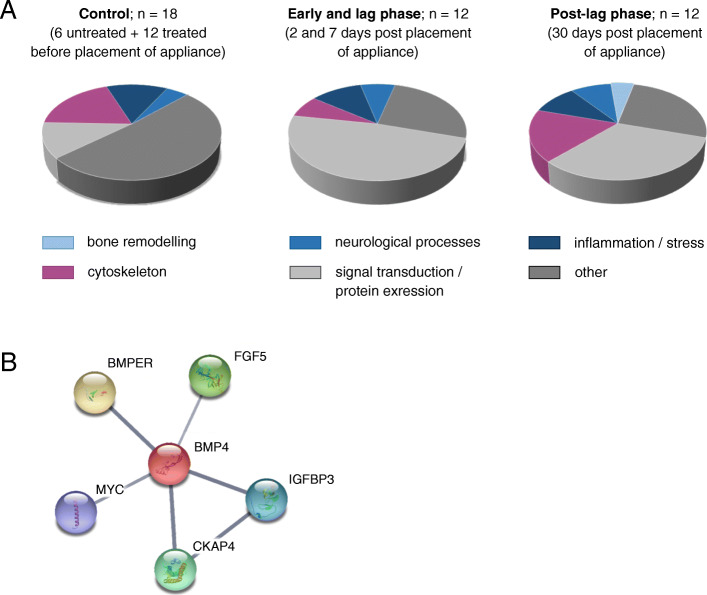
Saliva protein expression after fixed orthodontic apparatus placement. **A** Left panel shows proteins expressed in the control samples, which include pre-placement sample pools (n = 12), and control samples (n = 6) (no orthodontic therapy). The center panel presents proteins expressed in the early and lag phase of the OTM, which includes post placement (0 h), as well as two and then seven days post therapy (n = 12). The right panel shows proteins expressed in the post-lag phase of OTM at 30 days post placement when bone remodeling occurs (n = 12). Proteins are grouped according to the physiological processes they are part of. **B** Proteins interaction network related to saliva BMP4 in the post-lag phase, as predicted by STITCH software, version 5.0 (http://stitch.embl.de). Line thickness indicates strength of data support. BMP4 - bone morphogenetic protein 4; BMPER - BMP-binding endothelial regulator protein; FGF5 - fibroblast growth factor 5; IGFBP3 - insulin-like growth factor-binding protein 3; CKAP4 - cytoskeleton-associated protein 4; MYC - myc proto-oncogene protein

All participants were Caucasian and of European descent, which was not regulated by the inclusion/exclusion criteria. A total of 198 proteins were identified across all saliva samples (please see Supplementary Table S2 and Supplementary figure S1), which is in accordance with similar studies on the saliva [23]. They were classified into several functional subsets (Fig. 2A), but a subset of “bone remodeling-related” proteins was observed only in samples taken at 30 days post-placement of the appliance (Fig. 2A). The sample included 6 differential proteins (Table 2) with BMP4 depicted as a central protein in the interaction network (Fig. 2B). ELISA analysis suggested a detectable level of BMP4: 9.69 pg/mL in the purified/enriched samples corresponding to 0.32 pg/mL in unenriched, raw samples. Western blot (using purified, enriched samples) indicated a gentle visible band MW between 25 and 39 kDa in the saliva 30 days following the placement of fixed orthodontic appliance (as opposed to samples taken prior to and 7 days after the placement) which could correspond to BMP4 (see Supplementary methods 3).

**Table 2:** Protein interactions identified by STICH version 5.0 related to bone morphogenetic protein 4

Protein name	Peptide sequence	UniProt ID	Gene ID
Bone morphogenetic protein 4	RRPQPSK	H0YMP9	BMP4
BMP-binding endothelial regulator protein	VKLRAHR	Q8N8U9	BMPER
Fibroblast growth factor 5	IPLSAPR	P12034	FGF5
Insulin-like growth factor-binding protein 3	HRLAAGR	C9JMX4	IGFBP3
Cytoskeleton-associated protein 4	LFVKVEK	Q07065	CKAP4
Myc proto-oncogene protein	QRRNELK	H0YBT0	MYC

## Discussion

Periodontal tissues respond to OTM with a distinct pattern. Upon placement of the fixed orthodontic appliance, mechanical disruption of the periodontal ligament allows for some tooth movement. However, a lag phase follows in which the strained tissues get inflamed and no movement occur for several weeks. Finally, as the inflammation subsides, active bone remodeling takes over through expression of specific molecules involved in periodontal regeneration and homeostasis [1, 24]. Understanding the molecular mechanisms of OTM will advance the development of better appliances that maximize effect and minimize tissue damage. BMPs are major regulators of bone formation, and BMP4 expression in the saliva 30 days after orthodontic appliance placement indicates its possible role in alveolar bone formation and maintaining tissue homeostasis during dental displacement. Saliva BMP4 expression was confirmed by ELISA in a pooled and heparin-enriched sample 30 days after fixed orthodontic appliance placement.

The activity of BMPs is strongly influenced by developmental modulators like chordin, which prevents their activation [25]. The expression of BMPER or CV2, a chordin paralog with a clear inhibitory effect on BMP molecules, has also been confirmed for the first time during OTM in this study [26]. Recent studies show that BMPER can have antagonistic or agonistic effect on BMP molecules, depending on the relative concentration of BMPER and its ligands [26]. BMPER is expressed in the dental epithelium during development [27] where it promotes blood vessel formation by downregulation of antiangiogenic factor trombospondin-1 and upregulation of FGF2 and FGF receptor-1, potent angiogenesis promotors [28]. FGF2 is the most studied growth factor of the FGF family that is expressed in many tissues and body fluids, including the tears and saliva [29]. It induces periodontal regeneration and suppresses inflammation indicating its role in regeneration and healing [30]. In this study, FGF5 was also identified, which has the same effect on growth/differentiation balance of PDL cells, and is expressed in large amounts in PDL cell culture [31]. It has also been proposed as a regulator of BMP4 since FGF5 overexpression leads to decrease in BMP4 mRNA [32].

In OTM, depending upon its concentration, BMPER could inhibit or enhance new bone formation induced by BMP4. Additionally, it could affect the dental environment by inducing angiogenesis through the regulation of FGF molecules already identified in the saliva. Since both proteins have been identified in the saliva at the same time point, these results confirm a possible established BMP4/BMPER axis and shed new light on its role in bone remodeling during orthodontic therapy.

Another important bone metabolic pathway identified in this study is related to insulin growth factor-1 (IGF1). IGF1 induces mesenchymal to osteoblast differentiation and is highly expressed in bone matrix [33]. It is exclusively produced in the liver, from where it shortly circulates in the blood [34], and then rapidly binds to insulin-like growth factor-binding proteins (IGFBPs). IGFBP3 is the most abundant IGFBP that controls the distribution and activity of IGF-1 through interaction with its receptors. IGFBP3 also binds to type I collagen and thus plays a role in the storage of IGF1 in the bone matrix. IGFBPs are processed by the BMP1 metalloproteinase, which also releases BMP2 and BMP4 from their latent complexes. This processing also enhances some IGF-independent functions of IGFBP3 [35], including its inhibition of osteoblast differentiation through BMP2 signaling pathway suppression.

IGFBP3 is thus weaved into bone remodeling molecular networks arising during OTM. Our detection of IGFBP3 at day 30 of OTM could be related to the repair of the resorption lacunae during alveolar bone remodeling and root cementum repair. Saggese found a five-fold increase of IGF1 and IGFBP3 expression in the GCF of young patients from 8 to 15 years 1 month following orthodontic tooth movement [36].

BMP4 appears to be an important regulator of odontogenesis and tooth homeostasis. In tooth development, BMP4 is expressed in the dental follicle where it plays an important role in early tooth, alveolar bone, and soft tissue development [37]. Its expression levels remain high in postnatal bone remodeling, maturation, and homeostasis [12, 38, 39]. Increases in BMP4 expression occur in bone fractures, but importantly also during mechanical stress, and strain [17, 40]. Recent studies have found a specific mutation of the Bmp4 gene in patients with dental agenesis, which may be associated with the early onset of osteoporosis and osteopenia in humans [41]. Furthermore, BMP4 conditional knockout (BMP-4cKO) mice have a complete downregulation of dentin matrix acidic phosphoprotein 1 (DMP1) mRNA in cementoblasts, which is crucial for proper bone and dentin mineralization, and whose absence leads to periodontal breakdown and increased susceptibility to bacterial infection [42, 43].

BMP4 induced increased expression of cytoskeleton-associated proteins in the pluripotent stem cells [44] and, in vivo, showed odontogenic potential by inducing complete tooth formation from embryonic stem cells [45].

To the best of our knowledge, a BMP molecule was, up to now, never detected in the saliva, which raises important new questions. What is the exact role of and mechanism of action of BMP4 in alveolar bone remodeling? It also remains to be elucidated if the detected BMP4 stems locally from the GCF or does it originate from the blood, sourced from the carotid artery.

This pilot proteomic study has three main limitations. A relatively small sample size potentially does not fully represent the OTM proteome of the saliva. Therefore, only prominent bone remodeling regulators might have been identified, while weaker, but possibly important modulators might have been omitted. The second limitation refers to the time points of the study, since prolonged monitoring of the OTM saliva proteome over several months might accentuate or abate the seen effects. The third limitation is analyzing pooled samples and the lack of interindividual variability assessment: namely, saliva samples taken from boys treated with a fixed orthodontic appliance (as well as those of the control, non-treated boys) at different time points were pooled by the time-point, hence inter-individual variability of the proteomic signals could not be quantified. Considering the nature of BMPs, it is expected that their levels in small volumes of biological fluids (in this case saliva) are below the detection thresholds, so this step was practically unavoidable. However, this was a pilot study based on the “shotgun” approach, a powerful method of context-dependent signaling mechanism discovery [46]; a number of sources of variability in proteomic saliva analysis (sex, age, oral hygiene, children—non-smokers) were controlled for by inclusion criteria; all samples were processed in the same way; and finally, control, preplacement, and early post-placement samples returned practically no signal for BMP4 and the entire cluster of “bone remodeling proteins” theoretically indicating an infinite difference between the “later lag” and “preplacement & early lag” (i.e., between “some” vs. “none”). Finally, the presence of the molecule placed into the center of the “bone remodeling network” (BMP4) was confirmed by Western blot and ELISA. Under these circumstances, the measured concentration and the fact that purification of the samples were needed in order to establish technical pre-requisites for immunological identification of the protein are of secondary and only illustrative relevance: the fact that it could be quantified offers additional proof that this finding is not merely an artifact. Hence, despite these limitations, we believe that the present data accurately identify a biological phenomenon and highlight the need for further research into the role of BMP molecules in tooth physiology. Additionally, inherent to the study design with only boys included (purposely, to exclude gender as a source of variability and bias in this pilot study), no potential gender differences could be observed, which should be addressed in further research which will include a larger number of both male and female participants and assess interindividual variability, which will then also be correlated with periodontal measurements and examinations.

The majority of OTM occurs in the post-lag phase as a direct result of bone remodeling due to mechanical pressure of the fixed orthodontic apparatus. The need to simultaneously produce and dissolve bone tissue at opposite sides of a tooth, requires strict spatial and temporal regulation, possibly mediated by the BMP family proteins (Fig. 1). This research indicates that mechanical force could initiate a cascade of events similar to embryonic tooth development. As BMP molecules are produced locally in small amounts (on demand), they are difficult to identify in biological fluids. Mechanical forces generated during OTM have perhaps allowed us to overcome its detection level in the saliva. BMP4 and its protein networks are thus emerging as important players whose role should be further studied in bone remodeling during OTM and in homeostasis.

## Conclusions

Being aware of the discussed limitations, we consider it justified to state that the present proteomic pilot study is the first to identify BMP4 protein, BMP antagonist BMP binding endothelial regulator (BMPER) or cossveinless-2 (CV2), and several other growth factors related to bone metabolism in the human saliva during the post-lag phase of OTM induced by placement of orthodontic appliances. To date, there is no published data on the presence of BMP molecules or their antagonists in the saliva or the gingival cervical fluid (GCF), regardless of different orthodontic treatments or diseases.

## Supplementary Information

**Additional file 1: Supplementary figure S1**. Venn diagram of salivary proteins expressed in pooled control and orthodontic tooth movement (OTM) samples. Identification of bone morphogenetic protein 4 in saliva after placement of fixed orthodontic appliance

**Additional file 2: Supplementary Methods 1**. Liquid chromatography and mass spectrometry.

**Additional file 3: Supplementary Methods 2**. HiTrap Heparin HP column purification and BMP4 ELISA

**Additional file 4: Supplementary Methods 3**. SDS-PAGE and Western blot analysis of BMP4.

**Additional file 5: Supplementary table S1**. Participants’ malocclusion types. In Class 1 malocclusion, the bite is normal, but the upper teeth slightly overlap the lower teeth. Class 2 malocclusion (retrognathism or overbite), occurs when the upper jaw and teeth severely overlap the bottom jaw and teeth. It is subdivided into division 1 where the incisors are proclined and division 2 where the incisors are retroclined

**Additional file 6: Supplementary Table S2**. Proteins identified across all saliva samples. Time points of control (C) and expreimental (E) sample pools are labeled -1, 0, 2, 7 and 30 denoting sampling times a day before, immediately after, and then 2, 7 and 30 days after the placement of the fixed orthodontic apparatus. BR - bone remodelling, NP - neurological processes, I/SR - inflammation / stress response, C - cytoskeleton, ST/PE - signal transduction / protein expression, O – other, U – unknown function.

## Data Availability

The datasets supporting the conclusions of this article are available in the ProteomeXchange Consortium via the PRIDE partner repository, PXD016481, http://proteomecentral.proteomexchange.org and additional datasets are included within the article (and its additional files).

## Abbreviations

BMP: Bone morphogenetic protein
BMP-4cKO: Bone morphogenetic protein 4 conditional knockout
BMPER: BMP-binding endothelial regulator
CKAP: Cytoskeleton-associated protein
CV: Crossveinless
DMP: Dentin matrix acidic phosphoprotein
ELISA: Enzyme-linked immunosorbent assay
FGF: Fibroblast growth factor
MS: Mass spectrometry
GCF: Gingival cervical fluid
HPLC: High performance liquid chromatography
IGF-1: Insulin-like growth factor-1
IGFBP: Insulin-like growth factor-binding protein
iBAQ: Intensity-based absolute quantification
LC-MS: Liquid chromatography–mass spectrometry
MYC: Myc proto-oncogene protein
OTM: Orthodontic tooth movement
PBS: Phosphate-buffered saline
PDL: Periodontal ligament
RT: Room temperature
SAS: Saturated ammonium sulphate
TGF-ß: Transforming growth factor ß
