# Supplementary figures and images for: Identification of bone morphogenetic protein 4 in the saliva after the placement of fixed orthodontic appliance
Source: Prog Orthod. 2021 Jul 12;22:19. doi: 10.1186/s40510-021-00364-6 (PMC8273045; doi:10.1186/s40510-021-00364-6)

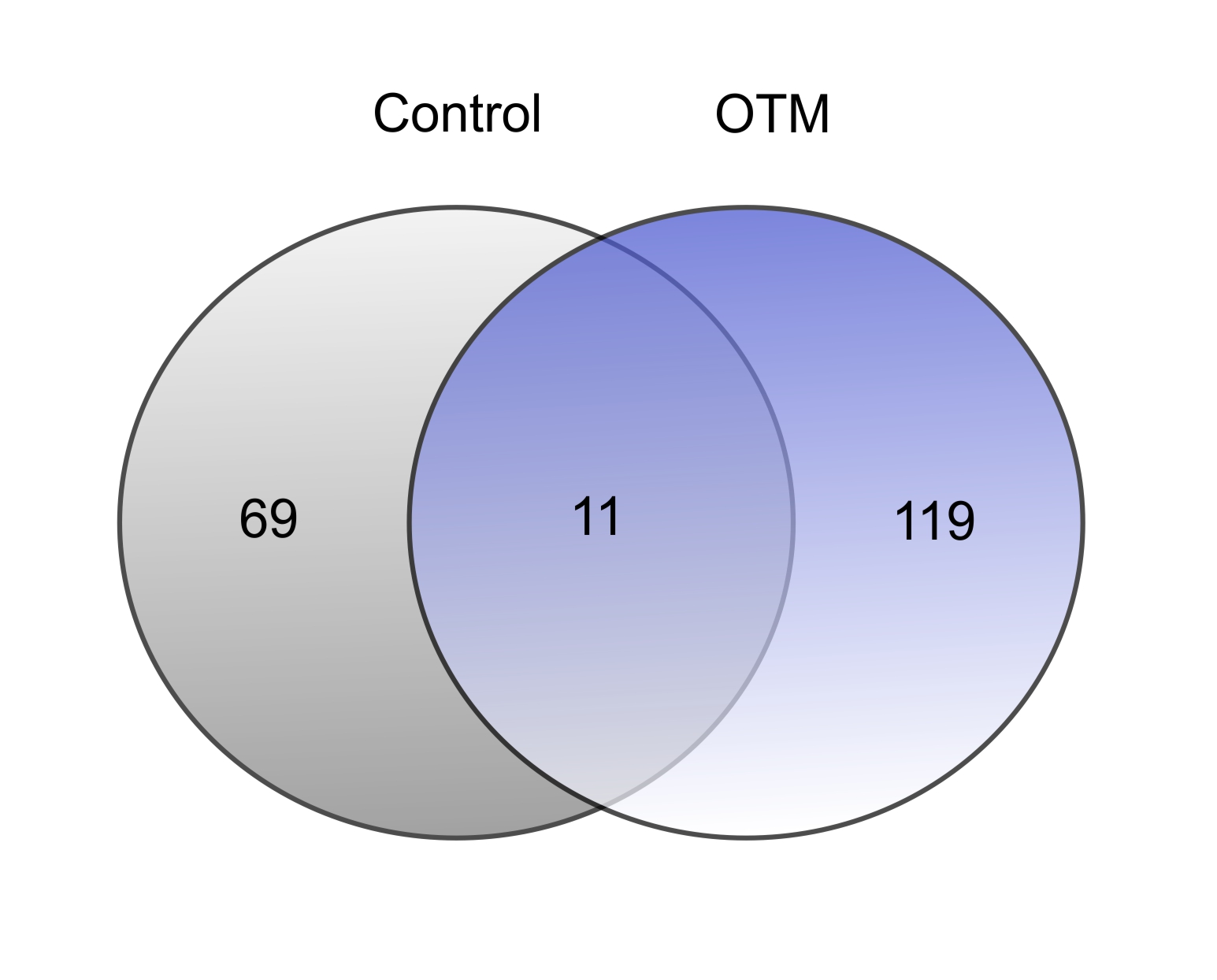

Supplement: Supplementary file 1 — Additional file 1: Supplementary figure S1. Venn diagram of salivary proteins expressed in pooled control and orthodontic tooth movement (OTM) samples. Identification of bone morphogenetic protein 4 in saliva after placement of fixed orthodontic appliance [file 40510_2021_364_MOESM1_ESM.jpg]
