# Supplementary material for: Identification of bone morphogenetic protein 4 in the saliva after the placement of fixed orthodontic appliance
Source: Prog Orthod. 2021 Jul 12;22:19. doi: 10.1186/s40510-021-00364-6 (PMC8273045; doi:10.1186/s40510-021-00364-6)
Supplement: Supplementary file 2 — Additional file 2: Supplementary Methods 1. Liquid chromatography and mass spectrometry. [file 40510_2021_364_MOESM2_ESM.docx]

Identification of bone morphogenetic protein 4 in saliva after placement of fixed orthodontic appliance

**Supplementary Methods 1- liquid chromatography and mass spectrometry**

Protein samples (40 µg) were transferred into 10 kDa centrifugal filter units, denatured using 8 M urea and alkylated for 20 minutes in dark at room temperature (RT) using 55 mM iodoacetamide in 8 M urea. After two washing steps (15 min, 14 000 g, RT) using 8 M urea, the samples were washed twice with 50 mM ammonium bicarbonate and digested overnight at 37 °C by 0.8 µg of trypsin (Worthington, TPCK treated). Digested peptides were eluted through 10 kDa filters into fresh Eppendorf tubes and then washed with 0.5 M sodium chloride. Peptides were then acidified using 20% acetic acid and separated by high performance liquid chromatography (HPLC) (Ultimate 3000, Thermo Fischer Scientific). A C18 micro pre-column and a 50 cm C18 nano-column were used to purify and separate peptides by polarity. Automated mass spectrometric measurement cycles (LTQ Orbitrap Discovery, Thermo Fischer Scientific) consisted of full mass spectrometry (MS) scanning and tandem mass spectrometry (MS/MS) scanning of ten most intense ions. Full MS scans ranging from m/z 300 to 2 000, were obtained in the Orbitrap analyzer at a resolution of 100 000, with internal calibration of the instrument using the lock mass setting. MaxQuant software version 1.5.1.2. (Max Planck Institute of Biochemistry) was used to process the raw data and quantify the detected proteins using intensity-based absolute quantification (iBAQ) algorithm. Trypsin was selected for in silico digestion, carbamidomethylation, N-terminal acetylation and methionine oxidation were used as variable peptide modifications. False discovery rate at the peptide spectrum level and at the protein detection level was set at 1%. Minimum peptide length for protein identification was seven amino acids. The main search peptide mass tolerance was set to 4.5 ppm. Common laboratory contaminants were excluded from the analysis. Proteins were quantified using iBAQ - a continuous intensity value of protein expression in individual samples (i.e. the ratio of the sum of the experimentally determined intensities of all peptides and the intensity of the individual detected peptide). Experimental data were compared with the set of human proteins available in the UniProt database (http://www.uniprot.org). Samples were analyzed in technical triplicates and proteins identified with at least one peptide were considered relevant for analysis [18]. The detected proteins were functionally classified according to their gene ontology, using the European Molecular Biology Laboratory - European Bioinformatics Institute (EMBL-EBI) Quick GO web based browser [19]. The mass spectrometry proteomics data were deposited at the ProteomeXchange Consortium via the PRIDE partner repository with the dataset identifier PXD016481. STITCH database version 5.0 (<http://stitch.embl.de>) was used to analyze interaction networks of proteins functionally classified as bone remodeling proteins.
