# Supplementary material for: Identification of bone morphogenetic protein 4 in the saliva after the placement of fixed orthodontic appliance
Source: Prog Orthod. 2021 Jul 12;22:19. doi: 10.1186/s40510-021-00364-6 (PMC8273045; doi:10.1186/s40510-021-00364-6)
Supplement: Supplementary file 3 — Additional file 3: Supplementary Methods 2. HiTrap Heparin HP column purification and BMP4 ELISA [file 40510_2021_364_MOESM3_ESM.docx]

Identification of bone morphogenetic protein 4 in saliva after placement of fixed orthodontic appliance

**Supplementary Methods 2 - HiTrap Heparin HP column purification and BMP4 ELISA**

Considering the expected very low BMP4 content in saliva samples (pooled samples of 12 subjects at 30 days post-placement of the orthodontic appliance), as is the case with BMP molecules in bodily fluids in general, saliva pool was first purified and enriched using a heparin column which has a high affinity

for BMP molecules (Figure A indicates heparin binding site in BMP molecules)


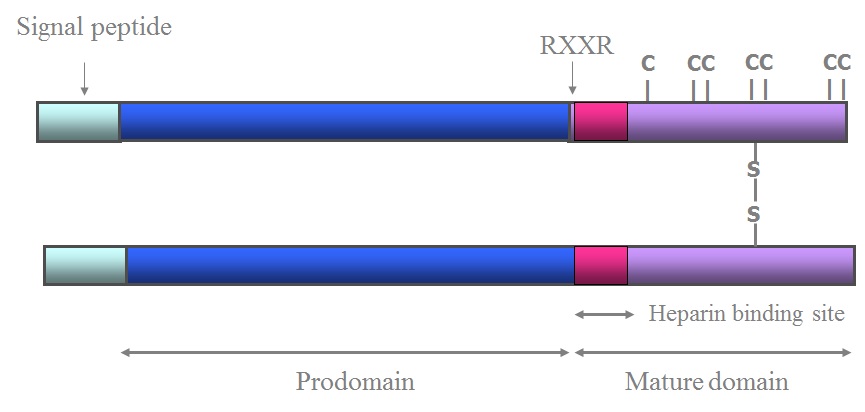


**Figure A**. General structure of a BMP molecule.

**Materials and methods**

- HiTrap heparin column HP (1 ml, # 17-406-01, GE Healthcare Biosciences AB SE-751 84 Upsala, Sweden)
- Saliva samples ( control, after 7 and 30 days after the placement of the fixed orthodontic appliance)
- Binding, washing and elution buffer
- Saturated ammonium sulphate

**HiTrap Heparin HP column purification (in this experiment the columns were operated with a syringe):**

- The samples were slowly thawed on ice (+4 °C).
- The samples were centrifuged at 14 000 x g for 5 minutes at +4 °C, for the precipitation of large particles.
- HiTrap heparin column HP (1 ml, # 17-406-01, GE Healthcare Biosciences AB SE-751 84 Upsala, Sweden) was equilibrated with 10 ml of 10 mM Na_2_HPO_4_.
- Total volume of 12 mL was passed through the heparin column. Flow-through was saved and passed for the second time to further enrich the column with BMPs.
- Heparin column was washed with 2 ml of 10 mM Na_2_HPO_4_ and the wash solution was saved for further processing_._
- BMPs were eluted from the column with 3 ml of 10 mM Na_2_HPO_4_ + 1 M NaCl and 3 ml of 10 mM Na_2_HPO_4_ + 2 M NaCl. Both eluates were collected into one tube.
- BMPs in the wash and eluate solutions were precipitated with ice cold 35% saturated ammonium sulphate for 90 minutes at +4 °C. Our previous research with BMP molecules showed 95% of BMP recovery after precipitation of BMP eluate fraction with SAS.
- After that, precipitated proteins were spun down at 15 000 x g for 15 minutes at +4 °C, and the supernatant was discarded.
- Precipitates from wash and eluate solutions were re-suspended in 200 µl of PBS each.
- 40 µl of each sample (wash and eluate) solution was taken away and stored for future Western blot analysis, in case such is needed.
- Concentration of the BMP4 protein in both solutions was measured using BMP4 ELISA R&D DuoSet (#DY314, R&D Systems, Inc. 614 McKinley Place NE, Minneapolis,
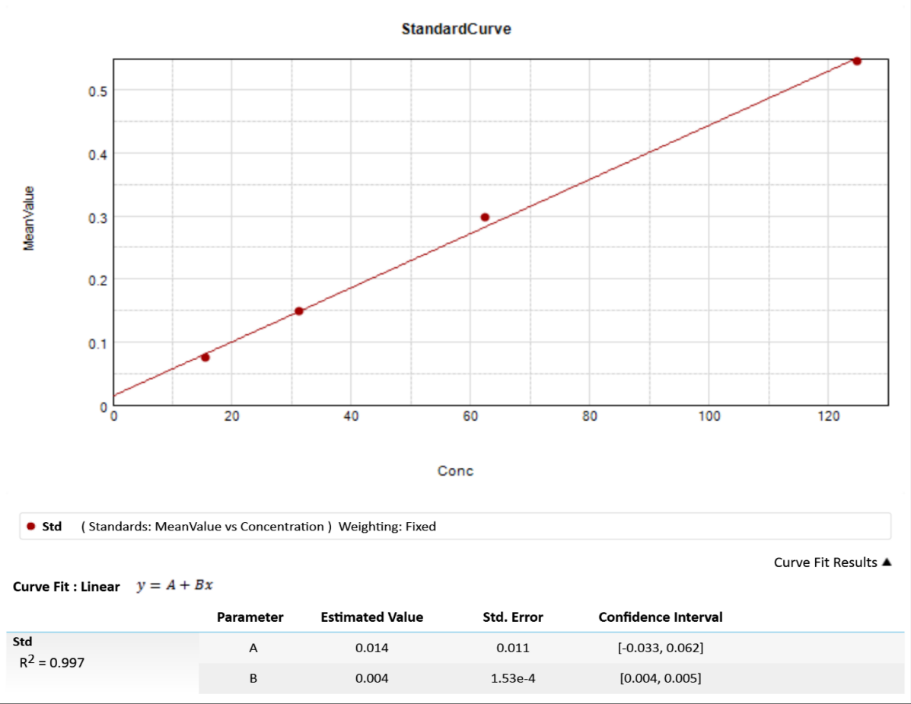
USA): 96-well plate was coated at 4 °C with 4 ng/ml mouse anti-human BMP4 capture antibody overnight. All of the following steps were done at room temperature and ELISA plate was washed with 0,05% tween 20 in PBS (PBST) three times between each step. After 60 minutes of blocking with 1% BSA in PBST, undiluted samples (wash and eluate) in duplicates and BMP4 standard (c=125, 62.5, 31.3 and 15.6 pg/ml) were incubated for 90 minutes. Following a 90-minute incubation with 180 µg/ml biotinylated mouse anti-human BMP-4 detection antibody. After 20 minutes of incubation with Streptavidin-HRP, color was developed with H_2_0_2_ + Tetramethylbenzidine (# DY994, R&D Systems) and the reaction was stopped with 2 N H_2_SO_4._ Optical density was determined using Spectra Max i3x microplate reader (Molecular Devices LLC. 3860 N First Street, San Jose,CA 95134, USA) at 450 nm with the correction for 570 nm. Results were analyzed using Soft Max Pro 7.0.3. Software (Molecular Devices LLC.).

**Results**

Absorbance values of BMP4 standards are shown in Table A and standard curve and curve fit are depicted in Figure B. BMP4 ELISA results for each sample well are shown in Table B.

**Table A.** Absorbance values of BMP4 standards.

| **Sample** | **ɣ (pg/ml)** | **BackCalcConc** | **Absorbance** |
| --- | --- | --- | --- |
| 1 | 125 | 123.532 | 0.545 |
| 2 | 62.5 | 66.029 | 0.298 |
| 3 | 31.3 | 30.983 | 0.147 |
| 4 | 15.6 | 13.855 | 0.074 |


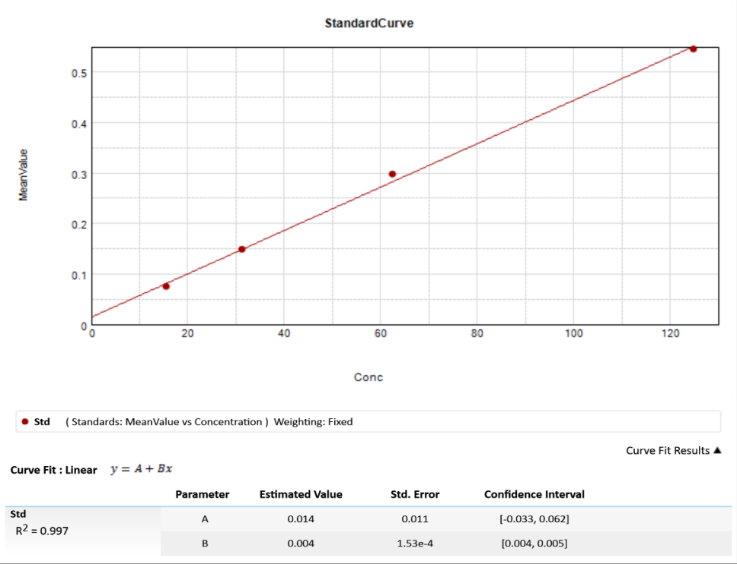


**Figure B**. Standard curve and curve fit, exported from the Soft Max Pro 7.0.3. Software

**Table B**. BMP4 ELISA results for each sample well.

| **Sample** | **Value** | **ɣ (pg/ml)** | **V (ml)** | **m (ɣ x V) (pg)** |
| --- | --- | --- | --- | --- |
| Eluate 1 | 0.037 | 5.291 | 0.08 | 0.42328 |
| Eluate 2 | 0.033 | 4.244 | 0.08 | 0.33952 |
| Wash 1 | 0.070 | 13.018 | 0.08 | 1.04144 |
| Wash 2 | 0.084 | 16.252 | 0.08 | 1.30016 |
|  |  | **TOTAL** | **0.32** | **3.1044** |

3,1 pg was detected in a total of 320 uL (40 uL was taken away in order to pursue Western-blot analysis), thus the concentration of the enriched sample (averaged for both wash and eluate) equates to 9.69 pg/mL.

A total of 12 mL of (raw) saliva was used for this analysis.
A total of 400 µL (200 in eluate and 200 in wash) was obtained after application to the heparin column.

Concentration factor: 12000 µL / 400 µL = 30

$$\frac{purified sample concentration}{concentration factor}=BMP4 concentration in saliva$$

*BMP4 concentration in saliva =* $\frac{9.69 pg/mL}{30}=0.323$ *pg/mL*

Estimated concentration: 0,32 pg/mL of BMP4 in a raw (unpurified) sample, i.e. in pooled saliva obtained from 12 children with fixed orthodontic appliances 30 days after the fixation.
