# Supplementary material for: Identification of bone morphogenetic protein 4 in the saliva after the placement of fixed orthodontic appliance
Source: Prog Orthod. 2021 Jul 12;22:19. doi: 10.1186/s40510-021-00364-6 (PMC8273045; doi:10.1186/s40510-021-00364-6)
Supplement: Supplementary file 4 — Additional file 4: Supplementary Methods 3. SDS-PAGE and Western blot analysis of BMP4. [file 40510_2021_364_MOESM4_ESM.docx]

Identification of bone morphogenetic protein 4 in saliva after placement of fixed orthodontic appliance

**Supplementary Methods 3 - SDS-PAGE and Western blot analysis of BMP4**

Purifed and enriched (for BMP content) (see Supplementary Method 1) pooled saliva samples obtained before placement (control), at 7 days after the placement and at 30 days after the placement of a fixed orthodontic appliance in 12 subjects (see Supplementary Method 1) were used in this analysis.

SDS-PAGE was run on a NuPAGE Novex™ 10% Bis-Tris Protein Gels, 1.0 mm, 10-well (Thermo Fisher Scientific) using MOPS SDS buffer system as instructed by the manufacturer. 30 μl of the sample were mixed with 10 μl of 4X LDS Sample Buffer (Lithium Dodecyl Sulfate buffer cat. no. NP0008, Invitrogen. Inc.) heated at 70⁰C for 10 min and loaded onto a NuPAGE™. A SeeBlue® Plus2 Pre-stained Protein Standard obtained from Thermo Fisher Scientific, (catalog #LC5925) was also loaded in a volume of 5 µl. Electrophoretic separation was done for 1 hour at 75 V constant voltages. The proteins were transferred by electro-blotting from the gel onto a nitrocellulose membrane. To avoid non-specific interactions of the antibody with the membrane, the membrane was subsequently immersed into 2% BSA solution. rhBMP4 was detected by a mouse monoclonal antibody specific for human BMP4 that is available as the capture antibody in the ELISA DuoSet kit (cat. no.#DY314 , R&D Systems).

**Results**

As shown in Figure A, Western blot indicates a gentle visible band MW between 25-39 kDa in saliva taken 30 days following the placement of a fixed orthodontic appliance which could correspond to BMP4.

| 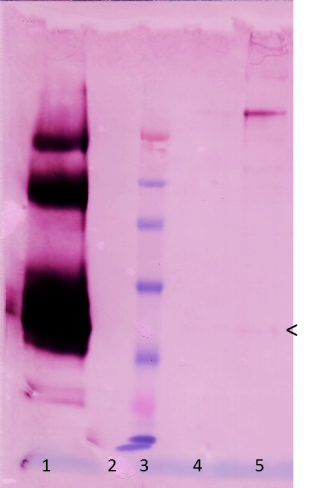 | **Figure A**. Western blot analysis of saliva samples (after heparin purification and enrichment) taken before (control), at 7 and at 30 days after placement of a fixed orthodontic appliance. Lane 1 rhBMP4 standard; Lane 2 control saliva sample; Lane 3 Rainbow; Lane 4 – sample taken 7 days after placement of the appliance; Lane 5 – sample taken 30 days after placement of the appliance. Arrow indicates a poorly visible band in Lane 5 MW between 25-39 kDa which could corresponds to BMP4. |
| --- | --- |
