# Supplementary material for: Identification of bone morphogenetic protein 4 in the saliva after the placement of fixed orthodontic appliance
Source: Prog Orthod. 2021 Jul 12;22:19. doi: 10.1186/s40510-021-00364-6 (PMC8273045; doi:10.1186/s40510-021-00364-6)
Supplement: Supplementary file 5 — Additional file 5: Supplementary table S1. Participants’ malocclusion types. In Class 1 malocclusion, the bite is normal, but the upper teeth slightly overlap the lower teeth. Class 2 malocclusion (retrognathism or overbite), occurs when the upper jaw and teeth severely overlap the bottom jaw and teeth. It is subdivided into division 1 where the incisors are proclined and division 2 where the incisors are retroclined [file 40510_2021_364_MOESM5_ESM.docx]

Identification of bone morphogenetic protein 4 in saliva after placement of fixed orthodontic appliance

**Supplementary table S1:** Participants’ malocclusion types. In Class 1 malocclusion, the bite is normal, but the upper teeth slightly overlap the lower teeth. Class 2 malocclusion (retrognathism or overbite), occurs when the upper jaw and teeth severely overlap the bottom jaw and teeth. It is subdivided into division 1 where the incisors are proclined and division 2 where the incisors are retroclined.

| **Cases** | **Malocclusion** | **Controls** | **Malocclusion** |
| --- | --- | --- | --- |
| 1 | Deep bite, Crowding, class II | Control 1 | Deep bite, class II division 1 |
| 2 | Deep bite class II division 2 | Control 2 | Deep bite, class I |
| 3 | Midline shift, class II | Control 3 | Severe crowding, class I |
| 4 | Deep bite, class I | Control 4 | Midline shift, class II |
| 5 | Crowding, class II div 2 | Control 5 | Cross bite, Hypodontic, class II |
| 6 | Deep bite class II | Control 6 | Deep bite class II |
| 7 | Severe crowding, class I |  |  |
| 8 | Deep bite, Crowding, class II |  |  |
| 9 | Deep bite, class II division 1 |  |  |
| 10 | Crowding, class II division 2 |  |  |
| 11 | Cross bite, Hypodontic, class II |  |  |
| 12 | Crowding, class II |  |  |
