# Supplementary material for: Identification of bone morphogenetic protein 4 in the saliva after the placement of fixed orthodontic appliance
Source: Prog Orthod. 2021 Jul 12;22:19. doi: 10.1186/s40510-021-00364-6 (PMC8273045; doi:10.1186/s40510-021-00364-6)
Supplement: Supplementary file 6 — Additional file 6: Supplementary Table S2. Proteins identified across all saliva samples. Time points of control (C) and expreimental (E) sample pools are labeled -1, 0, 2, 7 and 30 denoting sampling times a day before, immediately after, and then 2, 7 and 30 days after the placement of the fixed orthodontic apparatus. BR - bone remodelling, NP - neurological processes, I/SR - inflammation / stress response, C - cytoskeleton, ST/PE - signal transduction / protein expression, O – other, U – unknown function. [file 40510_2021_364_MOESM6_ESM.docx]

Identification of bone morphogenetic protein 4 in saliva after placement of fixed orthodontic appliance

**Supplementary Table S2.**

**Table S2**. Proteins identified across all saliva samples. Time points of control (C) and expreimental (E) sample pools are labeled -1, 0, 2, 7 and 30 denoting sampling times a day before, immediately after, and then 2, 7 and 30 days after the placement of the fixed orthodontic apparatus. BR - bone remodelling, NP - neurological processes, I/SR - inflammation / stress response, C - cytoskeleton, ST/PE - signal transduction / protein expression, O – other, U – unknown function.

| **Protein names** | **Peptide sequence** | **UniProt ID** | **Time point** | **Molecular process** |
| --- | --- | --- | --- | --- |
| 1-phosphatidylinositol 4,5-bisphosphate  phosphodiesterase beta-2 | EMEFLDITSIRDTRFGK | Q9BVT6 | E2 | ST/PE |
| 2-oxoglutarate and iron-dependent oxygenase domain-containing protein 2 | HFCRCACFCTDNLYVAR | F5H890 | E-1 | O |
| Actin-related protein 10 | LLGSTTPRR | G3V5Y4 | E30 | C |
| Adenomatous polyposis coli protein | ILKPGEK | P25054-2 | E30 | O |
| Adenylate cyclase type 9 | KKSSIQK | O60503 | E30 | ST/PE |
| A-kinase anchor protein 13 | HCTSTRK | H0YMW2 | C-1, C0 | ST/PE |
| Alcohol dehydrogenase 4 | GTKGKVIK | D6RIB1 | C-1, C7, C30, E-1, E0, E2, E7 | O |
| Alkylated DNA repair protein alkB homolog 1 | RDISTEGFCHLDDQNSEVK | Q13686 | C30 | I/SR |
| Alpha-amylase 1 | HMWPGDIK | Q5T085 | C-1, C0, C2, C7, C30, E-1, E0, E2, E7, E30 | O |
| Amyloid beta A4 precursor protein-binding family B member 1-interacting protein | PPPAVAK | Q7Z5R6 | C-1, E30 | C |
| Antigen KI-67 | PKTPLGK | P46013-2 | E30 | O |
| Aprataxin | GYVKVKQSLR | Q6JV79 | E7 | I/SR |
| Astrotactin-1 | EVAAGQVLKGTFR | B1AJS1 | E30 | NP |
| ATPase ASNA1 | YLDQMEDLYEDFHIVK | A0A087WXS7 | E-1 | O |
| ATP-dependent RNA helicase DDX18 | MSHLPMKLLRK | Q9NVP1 | E7, E30 | ST/PE |
| ATP-dependent RNA helicase DDX50 | LNGDTEEGFNRLSDEFSK | A0A087WVC1 | E2 | ST/PE |
| Autophagy-related protein 13 | EIKVSYTVYN | E9PPR2 | E30 | O |
| Beta-defensin 126 | PEEMHVKNGWAMCGKQR | Q9BYW3 | E-1 | I/SR |
| BMP-binding endothelial regulator protein | VKLRAHR | Q8N8U9 | E30 | BR |
| Bone morphogenetic protein 4 | RRPQPSK | H0YMP9 | E30 | BR |
| BRCA1-associated ATM activator 1 | PVSSWCTSWLCPCEVEPR | F8WDN5 | E-1 | I/SR |
| Bromodomain adjacent to zinc finger domain protein 2A | VPGRRGR | A0A0C4DGI9 | E30 | ST/PE |
| Calpain-6 | SLYLRKK | Q9Y6Q1 | C-1 | C |
| Calsyntenin-2 | VPDGIVPK | Q9H4D0 | C30 | NP |
| cAMP-dependent protein kinase type I-alpha regulatory subunit | ITRQWPL | X6RAV4 | E30 | ST/PE |
| Caspase-2 | PCTPEFYQTHFQLAYR | P42575-2 | E-1, E2 | I/SR |
| Chondroitin sulfate proteoglycan 5 | LSPASELPK | A0A087WUT8 | E2, E30 | NP |
| Cohesin subunit SA-3 | RRVEGPAK | H0Y4S6 | C30 | O |
| Complement C4-A | INVKVGGNSKGTLK | F5GXS0 | E30 | I/SR |
| Cyclic AMP-responsive element-binding protein 3-like protein 4 | SVLHADEM | Q8TEY5-2 | E30 | ST/PE |
| Cyclic nucleotide-gated cation channel beta-3 | LLWFKVK | Q9NQW8-2 | E30 | O |
| Cyclin-dependent kinase 8 | ITSEQAMQDPYFLEDP | A0A0D9SEP3 | E0 | ST/PE |
|  | LPTSDVFAGCQIPYPKR |  |  |  |
| Cytoskeleton-associated protein 4 | LFVKVEK | Q07065 | E30 | C |
| Cytosolic carboxypeptidase 1 | IIGPFSKK | J3KNS1 | E30 | C |
| Digestive organ expansion factor homolog | IIVSNKK | H7C2R4 | E30 | O |
| Disrupted in schizophrenia 1 protein | KTPLKVLEEWK | A0A087WYX6 | E30 | NP |
| DNA mismatch repair protein Msh2 | AGNKASK | C9J809 | C2 | I/SR |
| DNA transposase THAP9 | WELYNWRETDEYSAEMK | H0Y9F3 | C7 | ST/PE |
| DNA-3-methyladenine glycosylase | HVRSTLR | P29372 | E30 | I/SR |
| Docking protein 3 | GERRVIR | D6RAV2 | E30 | O |
| Down syndrome cell adhesion molecule | SQDVHIKAVLR | O60469-2 | E30 | NP |
| DPH3 homolog | EDLENGEDVATCPSCSLIIK | Q96FX2 | C30 | O |
| Dynein heavy chain 11, axonemal | PDLEKLK | A0A0C4DFR0 | E30 | C |
| Dynein heavy chain 17, axonemal | TPNVVEATSK | K7EK91 | C2 | C |
| E3 ubiquitin-protein ligase HERC2 | TPKLIEK | A0A0J9YXQ8 | E30 | I/SR |
| E3 ubiquitin-protein ligase MIB2 | PAELQRR | D6RED3 | E30 | NP |
| E3 ubiquitin-protein ligase TRIM69 | IKKLPLLK | A0A0G2JPI4 | C2 | O |
| E3 ubiquitin-protein ligase UBR4 | YDEDHSGDDKVFLDCFCK | X6R960 | C7 | I/SR |
| Early endosome antigen 1 | RNQQILK | Q15075 | E30 | C |
| EH domain-binding protein 1-like protein 1 | PEASGVDTEPRSGGREANTK | E9PIH6 | C30 | C |
| Enhancer of polycomb homolog | PLPIYRGK | E9PBA8 | E30 | ST/PE |
| Eukaryotic translation initiation factor 3 subunit D | TQKTAYQRNR | B0QYA8 | E30 | ST/PE |
| Eukaryotic translation initiation factor 4E type 1B | APGPLPK | H0Y8X3 | E30 | ST/PE |
| F-box only protein 47 | ELYCMDWTVKMMQKVCK | Q5MNV8 | E2 | O |
| FERM domain-containing protein 8 | DGTEGSAGQPGPAER | Q8N4M4 | E2 | I/SR |
| Fibroblast growth factor 7 | EGGDIRVR | H0YNE7 | C2 | C |
| Fibroblast growth factor 5 | IPLSAPR | P12034 | E30 | BR |
| Folate receptor gamma | QVNQSWRK | A0A087WYI3 | C2 | O |
| FYVE, RhoGEF and PH domain-containing protein 6 | TARLLRQK | F8VY01 | C-1 | C |
| Gamma-aminobutyric acid receptor subunit beta-1 | HGVPSKGRIR | P18505 | C7 | O |
| Glutamyl aminopeptidase | SVFDYFEEYFAMNYSLPK | Q07075 | E-1 | O |
| GPNMB protein | LLLYLSLK | Q96F58 | C2 | O |
| GRAM domain-containing protein 2B | FETPGSPR | D6REP5 | E30 | O |
| H/ACA ribonucleoprotein complex subunit 4 | PPLIAAVKR | H7BZF2 | C2 | O |
| Heterogeneous nuclear ribonucleoprotein L | RRSVPAR | M0R1W6 | E30 | ST/PE |
| Histone deacetylase 10 | SLWLNIR | C9J8B8 | C-1 | O |
| Histone-lysine N-methyltransferase SMYD3 | EPLKVEK | B0QZ88 | E30 | ST/PE |
| HSPB1-associated protein 1 | INVVNPDLKR | Q96EW2-3 | E30 | I/SR |
| Importin-9 | EVTDTQMPLVAPVILPE | Q96P70 | E0 | O |
|  | MYKIFTMAEVYGIRTR |  |  |  |
| Inactive rhomboid protein 2 | DSGCCVQNDHSGCIQTQRK | Q6PJF5-2 | C30, E2 | ST/PE |
| Inactive rhomboid protein 2 | LQSRKPPN | K7ELA9 | C30, E2 | ST/PE |
| Inactive ubiquitin carboxyl-terminal hydrolase 54 | EPSISSDTRTDSSTESYPYK | R4GN32 | C30 | O |
| Inhibitor of nuclear factor kappa-B kinase-interacting protein | KMEDLTMQMFNMEDDMLK | Q70UQ0 | C7 | O |
| Insulin-like growth factor-binding protein 3 | HRLAAGR | C9JMX4 | E30 | BR |
| Interactor protein for cytohesin exchange factors 1 | KFWVILK | E5RK08 | E30 | C |
| Interferon-induced protein with tetratricopeptide repeats 1B | EATNWQPRGQDRETVDR | Q5T764 | C7, E2 | I/SR |
| IQ motif and SEC7 domain-containing protein 1 | GVQYLIER | A0A0C4DGT3 | E30 | O |
| Janus kinase and microtubule-interacting protein 3 | MELLQLAQQRIKELEER | Q5VZ66-2 | C0 | C |
| Kalirin | EPLQLPK | C9IZQ6 | E-1 | O |
| Kanadaptin | KPALPVSPAAR | A0A087WWF4 | E30 | O |
| Kinetochore-associated protein 1 | RHPKLLAK | J3KQF2 | C-1 | C |
| Krev interaction trapped protein 1 | RDVRLPLEVEK | O00522-2 | E7 | O |
| Leucine-rich repeat and fibronectin type-III domain-containing protein 2 | LLVLEGQAATLK | Q9ULH4 | E30 | NP |
| Leucine-rich repeat serine/threonine-protein kinase 1 | GGARDLLEEACDQCASQLEK | E9PK39 | C7 | NP |
| Lymphocyte cytosolic protein 2 | PPIRAEGR | E7ESF6 | C30 | I/SR |
| Matrilin-2 | EDHNCEQLCVNVPGSF | E5RJM4 | E0 | NP |
|  | VCQCYSGYALAEDGKR |  |  |  |
| Metallothionein | CTSCKKSECEAISMVWGCG | H3BRY8 | E2 | O |
| Microtubule-actin cross-linking factor 1, isoforms 1/2/3/5 | APISPKK | E9PLY0 | E30 | C |
| Mitogen-activated protein kinase kinase kinase kinase 4 | NPPPRLKSK | I3L2I2 | C30 | I/SR |
| Mucin-16 | VFTSSIK | Q8WXI7 | C-1, C0, C2, C7, C30, E-1, E0, E2, E7, E30 | O |
| Multidrug resistance-associated protein 9 | GVQELKK | Q96J65-5 | E30 | O |
| Myc proto-oncogene protein | QRRNELK | H0YBT0 | E30 | ST/PE |
| Myosin light chain kinase 3 | PDPEPLEEGTR | Q32MK0 | E30 | O |
| Natriuretic peptides A | DGGALGRGPWDSSDRSALLK | B0ZBE8 | C30 | O |
| Nebulette | DLENEIKGK | O76041 | C2 | O |
| Nectin-3 | QTSSIAVAGAVIGAVL | Q9NQS3 | E0 | O |
|  | ALFIIAIFVTVLLTPRK |  |  |  |
| Neurotrypsin | HGSVRLR | P56730 | E30 | NP |
| Nidogen-2 | HAFCTDYATGFCCHCQSK | Q14112-2 | E2 | C |
| Nuclear receptor corepressor 2 | NRLLSPR | C9JQE8 | E30 | ST/PE |
| Nuclear receptor subfamily 2 group C member 2 | HGAEEKQNSQR | H7C3R1 | E30 | ST/PE |
| Nyctalopin | FSSLLSK | REV__O75165 | E30 | O |
| OTU domain-containing protein 7B | TLGSKLKK | Q6GQQ9-2 | C-1, C7, C30 | I/SR |
| Periodic tryptophan protein 2 homolog | LLMLHGQKLK | A0A096LPI6 | E30 | ST/PE |
| Peroxisome proliferator-activated receptor gamma | PGLLNVK | E9PFV2 | E30 | ST/PE |
| Phenylalanine--tRNA ligase beta subunit | IKAPVYK | Q9NSD9 | E30 | ST/PE |
| Phosphatidylinositol 4-phosphate 3-kinase C2 domain-containing subunit alpha | SITKLKTK | A0A0C4DGF9 | C-1, C7 | O |
| Phospholipase A2, membrane associated | FSNSGSR | P14555 | E-1 | I/SR |
| PiggyBac transposable element-derived protein 4 | KQIPNDLK | Q96DM1 | E30 | O |
| PIN2/TERF1-interacting telomerase inhibitor 1 | PDPGRRK | E5RGR1 | E30 | C |
| Probable ATP-dependent RNA helicase DDX60 | FAGDILK | Q8IY21 | E30 | O |
| Probable G-protein coupled receptor 101 | RQHALLYNVKR | Q96P66 | E30 | ST/PE |
| Probable phospholipid-transporting ATPase VA | FTPSCLTSGCSSIGSLAANK | O60312 | C30 | O |
| Proline-rich basic protein 1 | PRSPSPPR | E7EW31 | C-1, C0, C2, C7, C30, E-1, E0, E2, E7, E30 | O |
| Protein Aster-B | SHKRLSK | Q3KR37 | E30 | O |
| Protein bicaudal D homolog 2 | LKSLLSTK | U3KQF7 | C30 | C |
| Protein crumbs homolog 2 | SDPALYGGVQAAFPGAFSFR | Q5IJ48-2 | C7 | O |
| Protein FAM117A | GGAGGLR | D6RFX7 | E30 | U |
| Protein FAM50A | EREKQLAK | B0S8I6 | C30 | O |
| Protein kintoun | AEDGGHDTCVAGAAGSGVTT | Q9NVR5-2 | C7 | C |
|  | LGDPEVAPPPAAAGEERVPK |  |  |  |
| Protein lifeguard 2 | TQGKLSVANK | F8VZI9 | C7 | NP |
| Protein Mpv17 | FIPGTTK | C9J473 | E30 | O |
| Protein SCAF8 | PPISKAK | A0A0A0MT33 | E30 | ST/PE |
| Protein ZBED8 | LEDFWCAQFTAFPNLAK | Q8IZ13 | C7, E-1 | U |
| P-selectin | KALTNEAENWADNEPNNK | F6VVT6 | C7 | O |
| Putative deoxyribonuclease TATDN2 | QLQLAVSLKKPLVIHCR | H7BZJ2 | C7 | O |
| Putative exonuclease GOR | VGRQPGK | Q8IX06 | C0 | O |
| Putative FAM120A opposite strand protein | GPGPRPAR | Q5T036 | C30 | U |
| Putative inactive cathepsin L-like protein CTSL3P | GYVTPVK | Q5T8F0 | E30 | O |
| Putative olfactory receptor 10D3 | ITISILSIRTTEGR | Q8NH80 | C7 | O |
| Putative Polycomb group protein ASXL3 | NLVTNVALPVK | Q9C0F0 | E0 | ST/PE |
| Putative uncharacterized protein ADORA2A-AS1 | PALLAPSLATLK | P86434 | E30 | U |
| Putative uncharacterized protein FLJ11871 | VRITGGK | Q9HAA7 | E30 | U |
| Putative uncharacterized protein FLJ44790 | VQPGRGK | Q6ZTC4 | C-1, C0 | U |
| Putative uncharacterized protein PNAS-138 | YSEFTLK | Q9BZS9 | C7, E30 | U |
| Pyrroline-5-carboxylate reductase | ISPAALK | A0A087WTV6 | C7 | O |
| Receptor protein-tyrosine kinase | YGNLSNFLRAK | E9PD35 | E30 | O |
| Regulator of G-protein signaling 10 | AVSRLSRK | O43665 | C30 | ST/PE |
| RelA-associated inhibitor | VLAEIPR | Q8WUF5 | E30 | ST/PE |
| Retinoblastoma-associated protein | STSLSLFYK | P06400 | C2 | ST/PE |
| Rho GDP-dissociation inhibitor 1 | GGRAVLQPR | J3QQX2 | C7, E-1 | O |
| Scavenger receptor cysteine-rich type 1 protein M160 | LVGGSSR | Q9NR16-3 | E-1, E30 | I/SR |
| Secernin-1 | QGLEAMEEILTSSEPLD | Q12765-3 | E0 | O |
|  | PAEVGDLFYDCVDTEIK |  |  |  |
| Seipin | IISTSSR | E9PR78 | C0 | O |
| Serine/threonine-protein kinase PAK 6 | LSVISSNTLR | H0YM99 | C2 | O |
| SH2 domain-containing adapter protein F | AFAVDIK | H0YN16 | E30 | I/SR |
| Signal transducer and activator of transcription 1-alpha/beta | TLQNRGSSSQNNRVAECH | D2KFR9 | C7 | O |
| Solute carrier family 12 member 3 | NRVKSLR | J3QSS1 | E30 | I/SR |
| Somatotropin | LEDGSPR | B1A4G9 | C-1 | O |
| Sorting nexin-22 | ASNWGTLR | Q96L94 | E30 | C |
| Spectrin beta chain, non-erythrocytic 5 | VVQERLR | Q9NRC6 | E30 | C |
| Splicing factor 3A subunit 3 | LFSTKGK | Q12874 | E-1 | ST/PE |
| Striatin-interacting protein 1 | RSILGLPPLPEDSIKVIR | Q5VSL9-3 | C7 | C |
| Synaptojanin-2 | GGKAGNK | E7ER60 | C2, C7, C30, E30 | C |
| Synaptotagmin-like protein 2 | TVVHPKVK | A0A0U1RRJ3 | C-1 | O |
| Syntaxin-17 | LEPAIQK | E9PJW1 | E30 | C |
| TBC1 domain family member 10A | FGFIVGSQGAEGAFVCGAKR | F8WDN6 | C30 | O |
| T-cell leukemia homeobox protein 2 | EERQALR | F1T0F2 | C-1, C0 | NP |
| T-complex protein 1 subunit theta | QITSYGETCPGLEQYAIK | P50990-3 | C7 | O |
| Teashirt homolog 1 | QPDSPAGSTTSEEKK | Q6ZSZ6-2 | E2 | ST/PE |
| Teashirt homolog 2 | ESPHEEASSFSHSEGDSFRK | Q9NRE2-2 | C30 | ST/PE |
| Thymidine kinase | SLTSWSSAR | K7ENW5 | C2 | O |
| Tigger transposable element-derived protein 7 | QKLSMIIK | Q6NT04-2 | C30 | O |
| Titin | EPVAIKK | A0A0A0MRA3 | E-1, E30 | C |
| T-lymphoma invasion and metastasis-inducing protein 2 | HGKEDTLRLLK | E9PMZ8 | E7, E30 | ST/PE |
| Trans-2,3-enoyl-CoA reductase-like | FHKACPK | D6RBZ3 | C0 | O |
| Transcription initiation factor TFIID subunit 1-like | FLHLFGPGK | Q8IZX4 | C-1 | ST/PE |
| Transmembrane protein 139 | PQELDQPPPYSTVVIPP | Q8IV31 | C7 | O |
|  | APEEEQPSHPEGSRRAK |  |  |  |
| Transmembrane protein 198 | SWALLALWPLL | C9JXI5 | E30 | BR |
| Transmembrane protein 43 | VPAKKLE | Q9BTV4 | E30 | O |
| Trinucleotide repeat-containing gene 6B protein | DEEPSGWEEPSPQSISRK | H0Y720 | E2 | ST/PE |
| Tripartite motif-containing protein 66 | FTDLLGQGPIVPGLDAP | B5MCJ9 | E0 | ST/PE |
|  | KDLAIPSELEEPINLSVKK |  |  |  |
| Trophinin | DVIQEYDEYFPEIIER | B1AKE8 | E2 | C |
| Tyrosine-protein phosphatase non-receptor type 23 | NAVRVPR | C9JD91 | E30 | C |
| U3 small nucleolar RNA-associated protein 14 homolog C | LLEAIISLDGKNR | Q5TAP6 | E30 | ST/PE |
| Uncharacterized protein C11orf53 | HTVKDLLAEKR | A0A1B0GU63 | E7, E30 | U |
| Uncharacterized protein C17orf85 | NRIGNKLPPEK | Q53F19-2 | E7, E30 | U |
| Uncharacterized protein C3orf86 | PALAPPSK | P0DN24 | C30 | U |
| Uncharacterized protein KIAA1671 | PEMGSWLGRDPPDMTKLK | Q9BY89 | C0, E-1 | U |
| Uncharacterized protein KIAA1958 | GNIPGRK | Q8N8K9 | E30 | U |
| Unconventional myosin-Va | VLLLHLEEGK | G3V3C9 | C7 | C |
| Uridine diphosphate glucose pyrophosphatase NUDT14 | LGLSHPI | F8VU44 | E30 | O |
| Vacuolar protein sorting-associated protein 13B | ALVNPVK | Q7Z7G8-2 | E30 | C |
| Vacuolar protein sorting-associated protein 53 homolog | APASYTK | Q5VIR6 | C-1 | C |
| V-type proton ATPase subunit G 2 | LKQATRR | O95670 | E30 | O |
| WD and tetratricopeptide repeats protein 1 | KGPGGGAPVR | Q8N5D0-5 | C-1 | O |
| WD repeat-containing protein 86 | GARKPGR | Q86TI4 | C0 | U |
| Zinc finger C3H1 domain-containing protein | KVKDGAK | O60293-2 | C-1 | O |
| Zinc finger C3H1 domain-containing protein | QQTKAWK | O60293-2 | C-1 | O |
| Zinc finger CCHC-type and RNA-binding motif-containing protein 1 | YGKVVKVTIMK | Q8TBF4 | E30 | ST/PE |
| Zinc finger protein 148 | KQIREPVDLQK | Q9UQR1 | E7 | ST/PE |
| Zinc finger protein 219 | RKPASPGR | Q9P2Y4 | C30 | ST/PE |
| Zinc finger protein 23 | AFSVKGK | P17027-2 | E30 | ST/PE |
| Zinc finger protein 257 | SSHLTQHK | Q9Y2Q1-4 | E30 | ST/PE |
| Zinc finger protein 277 | VQVAPPL | E7EW13 | E30 | ST/PE |
| Zinc finger protein 462 | FPCEFCGRAFSQGSEWER | H3BLX4 | E-1 | ST/PE |
| Zinc finger protein 524 | GRRPGGATSSNR | K7EP10 | E30 | ST/PE |
| Zinc finger protein 621 | ECGKAFKSSYDCIVHEK | C9JZC2 | E2 | ST/PE |
| Zinc finger protein 765 | RYVARHR | Q7L2R6 | E30 | ST/PE |
| Zinc finger protein 841 | PLDVVLTSGIPK | M0R0F3 | E0 | ST/PE |
